# FimH-based display of functional eukaryotic proteins on bacteria surfaces

**DOI:** 10.1038/s41598-019-44883-z

**Published:** 2019-06-10

**Authors:** Markus Chmielewski, Johannes Kuehle, Danuta Chrobok, Nicole Riet, Michael Hallek, Hinrich Abken

**Affiliations:** 1 Center for Molecular Medicine Cologne (CMMC), University of Cologne, Cologne, Germany; 2 Department I Internal Medicine, Medical Faculty, University of Cologne, Cologne, Germany; 3 RCI, Regensburg Center for Interventional Immunology, Chair Gene-Immunotherapy, University Hospital Regensburg, Regensburg, Germany

**Keywords:** Expression systems, Cytokines

## Abstract

The demand for recombinant proteins for analytic and therapeutic purposes is increasing; however, most currently used bacterial production systems accumulate the recombinant proteins in the intracellular space, which requires denaturating procedures for harvesting and functional testing. We here present a novel FimH-based expression system that enables display of fully functional eukaryotic proteins while preventing technical difficulties in translocating, folding, stabilizing and isolating the displayed proteins. As examples, Gaussia Luciferase (GLuc), epidermal growth factor (EGF), transforming growth factor-α (TGF-α) and epiregulin (EPRG) were expressed as FimH fusion proteins on the surface of *E. coli* bacteria. The fusion proteins were functionally active and could be released from the bacterial surface by specific proteolytic cleavage into the culture supernatant allowing harvesting of the produced proteins. EGFR ligands, produced as FimH fusion proteins and released by proteolytic cleavage, bound to the EGF receptor (EGFR) on cancer cells inducing EGFR phosphorylation. In another application of the technology, GLuc-FimH expressed on the surface of bacteria was used to track tumor-infiltrating bacteria by bioluminescence imaging upon application to mice, thereby visualizing the colonization of transplanted tumors. The examples indicate that the FimH-fusion protein technology can be used in various applications that require functionally active proteins to be displayed on bacterial surfaces or released into the culture supernatant.

## Introduction

Bacterial surface display of recombinant proteins has become an attractive strategy for a broad range of applications such as production of bioadsorbents^1^, generation of cellular biosensors^2^, development of novel vaccine platforms^3^, screening of antibody libraries^4^ and whole-cell biocatalysis^5^. Generally, the procedure requires the fusion of the protein-of-interest (POI) to a bacterial surface protein to display the POI on the surface of the genetically modified bacteria^6^. Several surface-anchoring motifs like LPP-OmpA, LamB, PhoE, ice nucleation protein (INP) and auto-transporter are employed as carrier proteins for crossing the bacteria membrane. Despite the successful approaches, several problems remain to be solved, including the substantially reduced functional activity of the displayed proteins. Compared with their soluble form, surface-anchored β-lactamase fused to the translation unit (TU) of an auto-transporter shows substantially reduced catalytic activities^7^. A similar experience was made when displaying sorbitol dehydrogenase^8^. A major problem in the use of auto-transporters arises from the tertiary structure of the passenger domains and the size of the central cavity that permits translocating only small proteins. It seems not only to be a matter of size since even the 62 amino acids protein aprotinin is not efficiently translocated through the outer membrane^9^. Translocation by auto-transporters is very sensitive to structure of the passenger proteins that consist of a β-strands backbone with at least 300 amino acids thereby substantially limiting the applicability to variety of potential cargos^10^. As an alternative approach, protein sequences derived from the major *E. coli* lipoprotein (Lpp) were fused to the N-terminus of the POI to direct the protein to the outer membrane^11,12^. The system consists of two key anchoring motifs; the Lpp-derived signal sequence at the N-terminus to target the fusion protein to the inner surface of the outer membrane, and the outer membrane protein A (OmpA)-derived transmembrane region to transfer the protein across the outer membrane^12^. Since its introduction by Ghrayeb and Inouye^13^ in 1984, the Lpp-OmpA display method is facing difficulties including the low expression rate and the insufficient translocation efficiency which may be due to steric hindrance and incorrect folding when anchoring in the outer membrane^14,15^. In Gram-negative bacteria the outer membrane generally acts as a barrier to restrict the protein export from the cell interior; only pilins, flagellins, specific surface enzymes, and a few bacterial toxins are transported across the outer membrane^16^. These natural display systems have the benefit of being optimized for transporting and folding protein units to build polymeric structures on the extracellular surface making the display system attractive for biotechnological applications. We here used the fimbriae protein FimH, the mannose-specific adhesin of the *E. coli* type-1 fimbriae, for the extracellular display of recombinant proteins. Type-1 fimbriae are composed of up to 3,000 copies of the subunit FimA, that form the pilus rod, as well as the subunits FimF, FimG and FimH building the distal tip fibrillum^17,18^. In initial studies, Pallesen and colleagues used the positions 225 and 258 within the FimH adhesin to display the preS2 domain of the hepatitis B surface antigen or an epitope from cholera toxin^19^. Both positions within the FimH protein proved to be suitable for the integration of peptides of up to 56 amino acids which could be produced, displayed on the cell surface and partially conserved the adhesive function of FimH^19^. Longer peptide or full length proteins displayed by FimH in that position were so far not reported. While short polypeptides used for vaccines could be displayed, the technique failed in functionally expressing large proteins like enzymes or cytokines. Here we identified alternative positions within the FimH protein to display larger proteins in a functionally active fashion. Based on the 3D modelling of *E. coli* type-1 pili^20^ we identified the N-terminus of the FimH domain on the fimbriae tip as a suitable integration site of a larger protein. As examples, we genetically linked Gaussia luciferase (GLuc) and human epidermal growth factor (EGF), tumor growth factor-a (TGF-α) and epiregulin (EREG), all ligands of the epidermal growth factor receptor (EGFR), to FimH. Expressed by transformed *E. coli*, the proteins conserved their functional capacities. In particular, GLuc-FimH displaying *E. coli* bacteria were tracked during colonization of syngeneic tumors in an immunocompetent mouse model of pancreatic cancer during a six week period without losing GLuc activity. Bacteria with surface displayed proteins can be used for screening purposes and, furthermore, can be released in a functionally active form by specific proteolytic cleavage making the strategy attractive for protein production without the need to disrupt the bacteria by harsh procedures.

## Results

### Display of recombinant proteins on the surface of gram-negative bacteria

**Figure 1:**
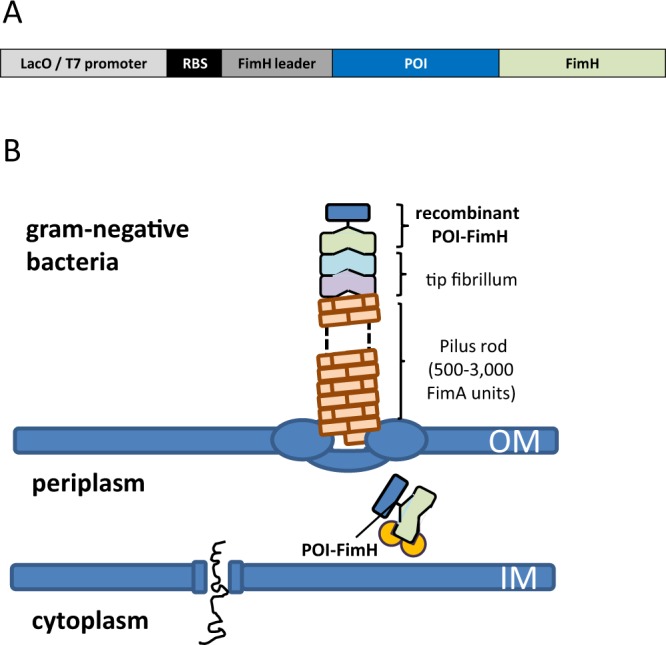
Schematic depiction of the POI-FimH fusion protein to display a recombinant protein on the surface of gram-negative bacteria. (**A**) Schematic depiction of the expression cassette used to express the protein of interest (POI) linked to the FimH protein. The protein is expressed upon IPTG-mediated de-repression of the T7 promoter and IPTG mediated induction of the T7 polymerase from the lacUV5 promoter in DE3 containing *E. coli* bacteria. (**B**) The POI is located at the top of the type-1 fibrillum N-linked to the FimH protein. OM, outer membrane; IM, inner membrane.

To enable translocation to the bacterial surface, proteins of interest (POI) were genetically fused to the N-terminus of the *E. coli* FimH adhesin. The FimH-fused POI cDNA was further linked to the FimH leader peptide by PCR, cloned into the pET-11d vector and transformed into *E. coli* strain BL21. Expression of the POI was induced by adding isopropyl-β-D-thiogalactopyranosid (IPTG), resulting in the display of the POI on the distal tip of the type-1 fibrillum (Fig. 1).

**Figure 2:**
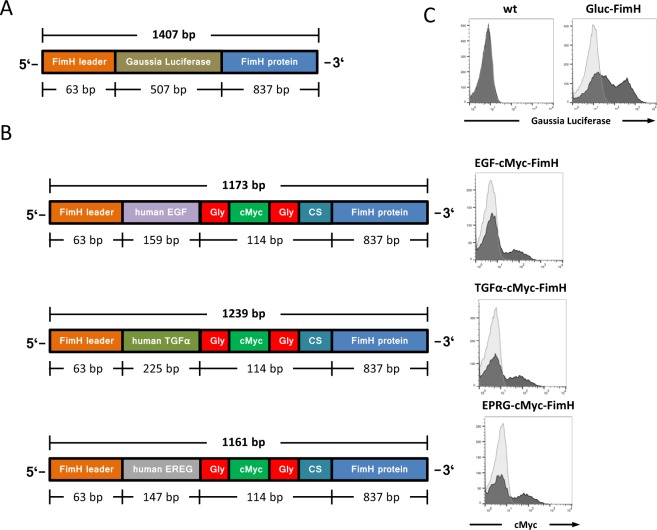
Design and expression of POI-FimH fusion proteins. (**A**) The Gaussia Luciferase (GLuc) is N-linked to the FimH protein and thereby becomes part of the fimbriae on the bacterial surface. By use of the bacterial pet11d vector, the expression of the GLuc-FimH fusion protein is induced by incubation with IPTG. (**B**) Schematic representation of the expression cassettes for the FimH fusion proteins with human EGF, human TGF-α and human epiregulin EPRG, respectively. Gly, Glycine-(Gly-Gly-Gly-Gly-Ser)_3_-linker; cMyc, c-Myc tag; CS, HRV3C protease cleavage site. (**C**) The expression of the POI-FimH protein on the surface of *E. coli* was induced by incubation with IPTG for 2 hrs and recorded by flow cytometry. Bacteria containing the GLuc-FimH expression vector were stained with a Gaussia luciferase-specific rabbit antibody and detected with a PE-coupled anti-rabbit antibody (bold histogram). An isotype-matched PE-conjugated antibody (light histogram) and non-modified *E. coli* (wt) served as controls. The expression of EGF-FimH, TGFα-FimH and Epiregulin-FimH on the surface of engineered *E. coli* bacteria was induced by IPTG. The bacteria were stained with a PE labeled c-Myc-specific mouse antibody and the proteins were recorded by flow cytometry (bold histograms). An isotype-matched PE-conjugated antibody (light histograms) served as control. All experiments were performed in triplicates and a representative histogram is shown.

Likewise, Gaussia luciferase (GLuc), epidermal growth factor (EGF), tumor growth factor-α (TGF-α) and epiregulin (EREG) were expressed as fusion proteins of FimH (Fig. 2A,B). Upon IPTG treatment the recombinant proteins were detected by flow cytometry using antibodies specific for GLuc or the c-myc tag incorporated into the POI-FimH fusion proteins (Fig. 2A).

**Figure 3:**
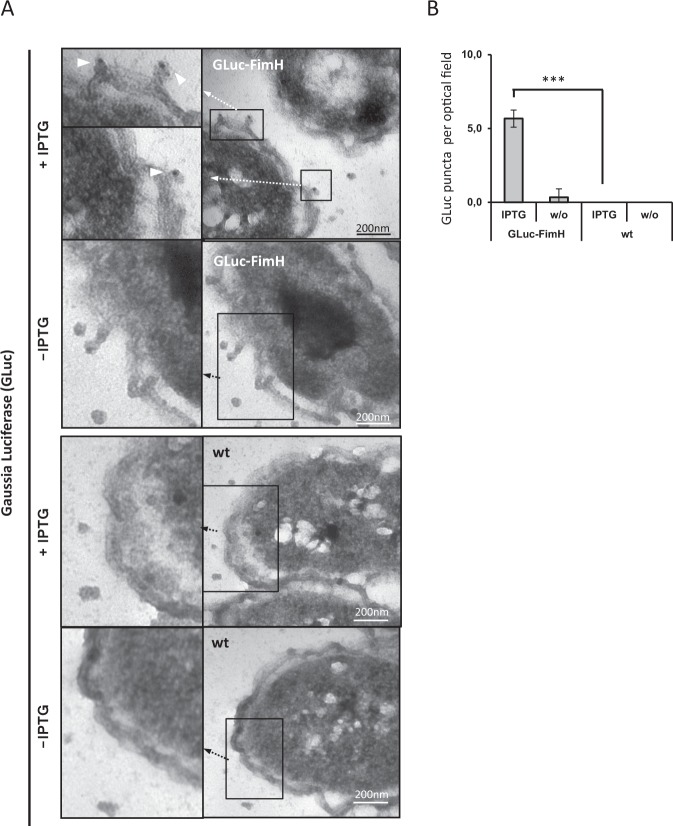
The GLuc-FimH fusion protein is located on the bacteria surface. (**A**) *E. coli* bacteria were transformed with the respective plasmid encoding the GLuc-FimH fusion protein. The expression was induced by incubation with IPTG; incubation without IPTG and non-modified (wt) *E. coli* served as controls. The samples were fixed and the expression of the GLuc-FimH protein on the bacterial surface was recorded by transmission electron microscopy using immunogold staining. White arrowheads indicate the presence of GLuc. (**B**) Number of colloidal gold labels on the surface of bacteria per optical field was quantified for GLuc-FimH (n = 10) engineered and wild-type bacteria (n = 10) in the presence or absence of IPTG; ***p < 0.001 (one-way ANOVA with Tukey’s multiple-comparisons test).

To visualize the recombinant GLuc-FimH as part of the fimbriae, the transformed *E. coli* bacteria were induced for expression by IPTG, fixed and subjected to immunogold staining with a rabbit anti-GLuc antibody and a colloidal gold-labeled anti-rabbit IgG antibody. As exemplarily shown in Fig. 3 (further examples in Supplementary Fig. S1), GLuc-FimH protein was specifically detected on the bacterial surface next to the outer membrane whereas control stainings with an isotype matched antibody of irrelevant specificity or staining of bacteria without IPGT induction did not detect GLuc protein.

### GLuc-FimH modified *E. coli* bacteria catalyze the conversion of the sGLuc substrate D-Luc

**Figure 4:**
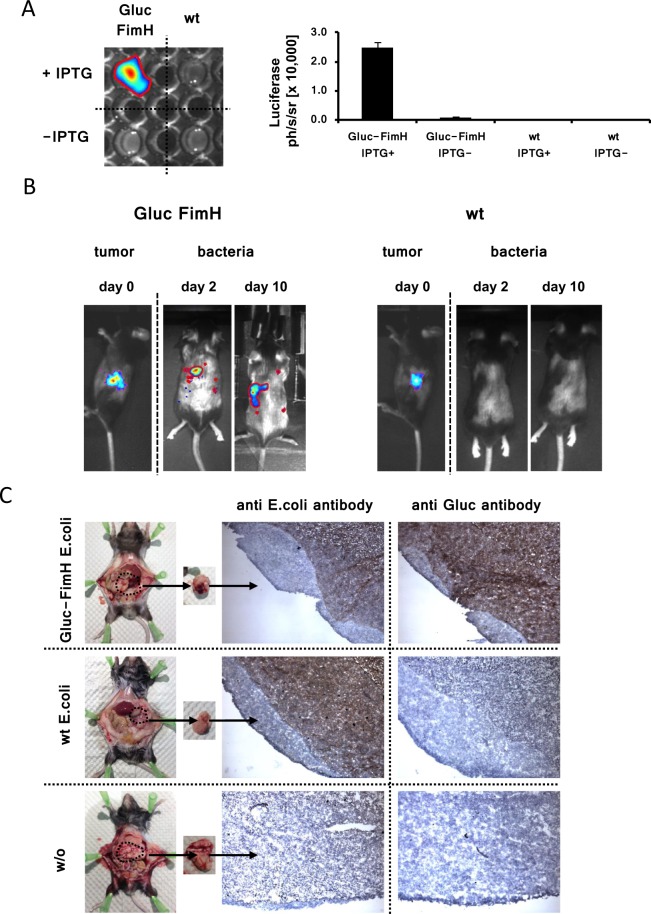
The GLuc-FimH fusion protein is functionally active on the bacteria surface. (**A**) *E. coli* bacteria were transformed with the plasmid encoding the GLuc-FimH fusion protein and tested *in vitro* for GLuc catalytic activity using benzyl-coelenterazine as substrate. Non-modified *E. coli* (wt) as well as GLuc modified *E. coli* bacteria without IPTG induction served as controls. Emitted photons [ph/s/sr] were recorded by the Photon Imager bioluminescence device; the signal intensity is displayed. All experiments were performed in triplicate wells for each condition and repeated at least twice. Data represent means ± s.d. of three replicate wells. (**B**) Click-beetle luciferase (CBL)-positive syngeneic pancreatic adenocarcinoma were established in immunocompetent mice by inoculation of Panc02 tumor cells. The accumulation of GLuc engineered *E. coli* bacteria in Panc02 tumors was analyzed by bioluminescence recording at day 0 and day 10 after bacteria inoculation. Spots indicate the accumulation of GLuc bacteria at the tumor site and at regional lymph nodes whereas the body was cleared from bacteria. (**C**) Pancreatic adenocarcinoma from mice after inoculation with Gluc-FimH engineered or wild-type *E. coli* were investigated for the presence of *E. coli* antigens and GLuc by immune histology staining using anti-*E. coli* and anti-GLuc antibodies. Pancreatic tumors derived from mice without bacteria inoculation served as controls.

To assay the functional capacity of the GLuc-FimH fusion protein, engineered *E. coli* bacteria were cultivated in the presence of IPTG and incubated with the GLuc substrate benzyl-coelenterazine for bioluminescence imaging (Fig. 4A). Transformed bacteria converted the GLuc substrate and emitted photo-detectable luminescence indicating enzymatic catalysis by the surface-displayed FimH-fused Gaussia luciferase. In contrast, neither GLuc-FimH modified bacteria without IPTG induction nor wild-type *E. coli* bacteria converted the GLuc substrate.

Bacteria preferentially accumulate and amplify at sites of immune repression including larger solid tumor lesions^21^. We here used GLuc-FimH bacteria to track their accumulation within established pancreatic carcinomas in immune competent mice. Therefore, cells of pancreatic cancer cell line Panc02 modified with click beetle Luciferase (CBLuc) for bioluminescence imaging were transplanted into the pancreas of syngeneic immunocompetent C57BL/6 mice. Transplanted carcinoma cells gave rise to a pancreatic adenocarcinoma that progressively increased in size (Fig. 4B). When tumors were established, i.e., reached a size of about 75 mm^3^, GLuc-FimH *E. coli* bacteria or control non-modified *E. coli* bacteria were injected into the tail vein of tumor bearing mice. GLuc-mediated bioluminescence was recorded on day 2 and day 10 after bacteria application using the GLuc specific substrate. GLuc-mediated bioluminescent signals were detected particularly at the tumor site of GLuc-FimH bacteria inoculated mice. GLuc-bioluminescence was also detected in regional lymph nodes which are likely due to tumor-associated macrophages transporting phagocytosed bacteria into regional lymph nodes (Fig. 4C). Bioluminescence data were confirmed by immunohistochemical stainings of the tumor tissues; GLuc-FimH engineered bacteria were detected in tumor tissues of treated mice, but not in untreated mice, using *E. coli*- and GLuc-specific antibodies (Fig. 4D). As expected, wild-type *E. coli* bacteria also colonized the tumor lesions as recognized by the *E. coli* specific antibody but not by the GLuc-specific antibody. No *E. coli* bacteria were found in pancreatic tumors of control mice without prior bacteria inoculation.

### Recombinant human EGF receptor ligands produced by transformed bacteria mediate EGFR phosphorylation

**Figure 5:**
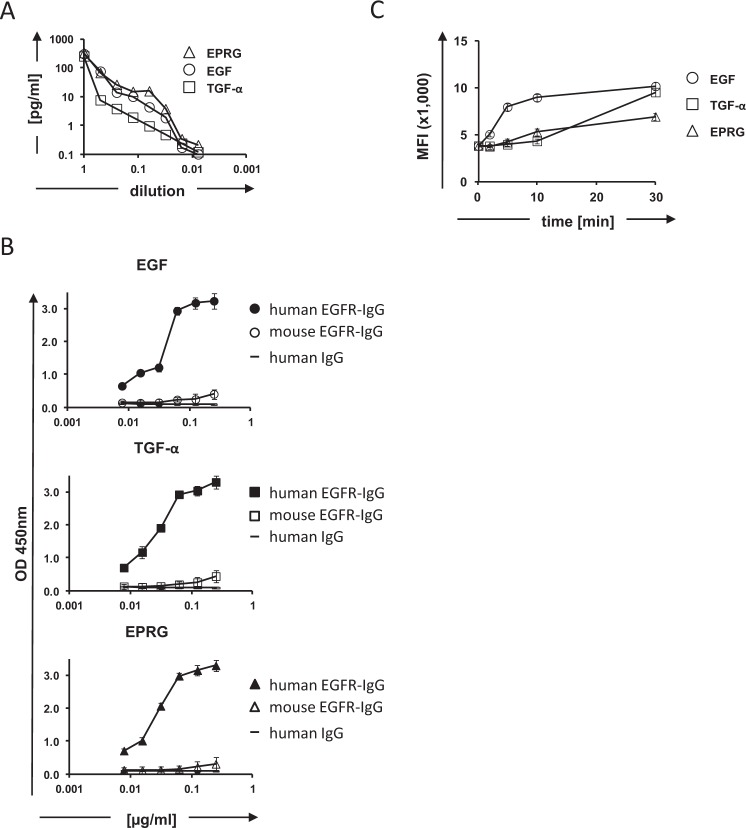
Recombinant EGFR ligands produced by transformed *E. coli* induce EGFR receptor phosphorylation. (**A**) *E. coli* bacteria were engineered with FimH fusion proteins linked with the EGFR ligand epiregulin, EGF or TGF-α, respectively. Displayed EGFR ligands were proteolytically released from the bacteria surface and detected by ELISA. The analysis was performed in triplicates for each condition and the entire experiment repeated at least twice; representative data are shown. (**B**) Proteolytically released proteins of interest, i.e., EGF, TGF-α and epiregulin were tested for binding to the EGF receptor (EGFR). Therefore, microtiter wells were coated with the bacteria produced EGF, TGF-α and epiregulin, respectively, and incubated with the human EGFR-hIgG (filled symbols) and mouse EGFR-IgG (open symbols) fusion protein to test for specific binding. A human IgG fusion protein of irrelevant specificity (bold line) served as control. Bound EGFR-IgG fusion protein was detected by a biotin-labeled human IgG-specific antibody. (**C**) EGFR^+^ 293T cells were incubated in the presence of proteolytically released EGF, TGF-α or EPRG, respectively, and tested for EGFR (Tyr1173) phosphorylation by flow cytometry. The mean fluorescence intensity (MFI) after incubation with the EGFR ligands is shown. All experiments were performed in triplicates for each condition and repeated at least twice; a representative experiment is shown.

To allow harvest of FimH-fused proteins we modified the POI-FimH fusion protein by inserting a cleavage site for the human rhinovirus 3C protease (HRV3C) between the C-terminus of the POI and the N-terminus of FimH (Fig. 2B). *E. coli* were transformed with the respective plasmids encoding the EGFR ligands EGF, EPRG and TGF-α linked to FimH, and induced for protein expression by IPTG. The proteins were detached from the bacteria surface by HRV3C protease digestion. To harvest the cleaved EGFR ligands, protease-treated bacterial culture supernatant was concentrated by ultrafiltration. The produced EGFR ligands in the processed supernatants were determined by ELISA (Fig. 5A). To test for binding to the human EGFR receptor, ELISA plates were coated with produced ligands and incubated with recombinant human EGFR-IgG fusion protein that was dectected by biotin-labeled human IgG-specific antibody. All three released EGFR ligands bound specifically to the human EGFR receptor (Fig. 5B).

To test for the induction of EGFR signaling, 293T cells were incubated with supernatants containing ligands EGF, TGF-α or epiregulin (1 µg/ml) and phosphorylation of the EGF receptor was detected via flow cytometry by intracellular staining with an anti-pEGFR (Tyr1173) antibody. As summarized in Fig. 5C, the released recombinant proteins triggered EGFR Tyr1173 phosphorylation indicating EGFR signaling (Fig. 5C). EGF induced EGFR phosphorylation with a faster kinetic than the ligands epiregulin and TGF-α. Taken together, data demonstrate the functional biological activity of all three bacteria-produced EGFR ligands.

## Discussion

In this study, we describe a bacterial expression system using the bacterial fimbriae component FimH as a vehicle for bacterial surface display of various heterologous proteins while retaining their functional capacities. The FimH-mediated protein display is based on anchoring the protein-of-interest in the distal tip of the fibrillum, thereby avoiding steric hindrances. The display of large proteins on the bacteria surface is likely facilitated by specialized periplasmic assembly factors and chaperones that bind individual pilus subunits in the periplasm and deliver them safely to an assembly platform in the outer membrane, to which the pilus is anchored^22^. The FimH-assisted display concept takes advantage of the chaperone mediated transfer through the membrane and display of proteins of sizes up to 200 amino acids. The displayed proteins on the fimbriae are functional as exemplarily shown by FimH-fused Gaussia luciferase (GLuc) that converted the cognate substrate for bioluminescence imaging after application in mice.

Our strategy differs in various means from previous approaches. In 1995, Pallesen and colleagues used two interior positions within the adhesin unit of FimH for surface display of heterologous sequences^19^. A serious limitation of the described strategy is the small capacity of up to 56 amino acids that can be successfully inserted into this position^19^. In contrast, we inserted proteins of nearly 200 amino acids on the tip of the fimbriae without loss of function thereby overcoming the size limitation as reported by Klemm and Schembri^23^. In addition, displaying fully functional eukaryotic proteins on bacterial surfaces make new applications possible. This includes the production of proteins with enzymatic activity as shown by the extracellular expression of GLuc. Also functional ligand – receptor interactions can be initiated by the produced recombinant EGFR ligands EGF, TGF-α and EPRG. In fact, considering the current needs and possibilities in producing recombinant proteins in bacteria for various biotechnological applications the presented technological solution helps to overcome major obstacles as they currently occur with respect to efficient and stable display on bacterial surfaces. This includes optimal anchoring of the FimH carrier protein in the bacterial outer membrane. In previous studies, various anchoring motifs were identified, including OmpC, OmpX and BclA for surface display of recombinant proteins^24–26^. Other bacterial proteins used for anchoring, such as outer membrane proteins LamB (maltoporin), OmpA (outer membrane protein A) and PhoE (phosphateinducible porin), were found unsuitable for the display of larger recombinant proteins; however, the Lpp-OmpA display system as an exception allows display of larger proteins on the surface of *E. coli* bacteria^11^, e.g., the alkaline phosphatase (PhoA) protein^15^. However, the use of current anchoring motifs did not always allow efficient display of every recombinant protein on the bacterial surface. Despite the achieved progress, further developments are required to provide a reliable biotechnological solution for the realization of individual requests. One of the major obstacles is the outer membrane itself. In order to be displayed on the surface, the recombinant proteins produced in the cytoplasm must be translocated through the inner membrane, cross the periplasm, be inserted into the outer membrane and there correctly folded and displayed^27^.

The novelty of the present study is that the fimbriae protein FimH was used as a carrier protein for the display of entire eukaryotic proteins on the surface of *E. coli* bacteria. In this respect, the study goes beyond the aims of Klemm and Schembri^23^ and Pallesen^19^ displaying peptides.

The displayed GLuc present of the surface of *E. coli* bacteria is enzymatically active in converting the specific substrate indicated by biolumescence which can be used to track the bacteria during colonization of syngeneic tumors in an immunocompetent mouse model of pancreatic adenocarcinoma. Bacterial colonization of tumors as sites of local immune repression is mediated by the reticuloendothelial system of the host, by the tumor microenvironment and by the bacterial metabolism^28^. This explains why the bacterial colonization is limited to immunocompromised areas such as tumors and can be efficiently prevented by the endogenous immune system outside the tumor. In this context, bacteria tracking by anchored luciferase activity open new fields to understand the interactions and to use bacteria as vehicle to deliver diagnostic marker or therapeutic drugs to the tumor.

In a further example, we demonstrated the universal applicability of the technology by fusing the epidermal growth factor (EGF), tumor growth factor-alpha (TGF-α) and epiregulin (EPRG), respectively, to FimH (Fig. 2B). All these proteins are ligands of the EGF receptor. In order to test their activity on the EGFR receptor, the produced EGFR ligands were proteolytically released from the bacteria surface and added to EGFR^+^ tumor cells. Interestingly, all ligands induced Tyr1173 EGFR phosphorylation, however, EGF stimulated more efficiently than EPRG and TGF-α. The data confirms previous work by Faria and colleagues who used recombinant EGF, TGF-α, EPRG, and two further EGFR ligands produced intracellularly by *E. coli*^29^. In contrast to conventional production methods the FimH produced ligands were proteolytically released from the carrier protein FimH without the need to lyse the *E. coli* bacteria. Since the bacteria remain viable and intact they can be re-used for protein production. For example, the bacteria were cultivated and harvested in a closed circuit while the POI was proteolytically cleaved from the carrier molecule FimH without destructing the bacteria. The bacteria can then be returned to the production circuit ready for repeated POI production. After proteolytic cleavage the POI is correctly folded; the technique thereby avoids known obstacles associated with protein recovery using of harsh denaturants and subsequent cumbersome protein-refolding procedures.

The POI display on the tip of fimbriae can moreover be used to screen a protein display library for various properties or for binding similar to phage display library screening. By binding to immobilized POI ligands the engineered bacteria are bound and can be amplified and enriched by repetitive rounds of binding, washing and amplifications. Such bacteria display library screening may be as efficient as phage display library screening. Taking together the described technology has the potential to broaden the production and application of recombinant proteins for diagnostic and therapeutic purposes.

## Material and Methods

### Primary cells and cell lines

The mouse pancreatic cancer cell line Panc02 was cultivated in RPMI 1640 medium, 10% (v/v) FCS, penicillin (100 U/ml), and streptomycin (100 mg/ml). 293T (ATCC, CRL-11268) is a human embryonic kidney cell line that expresses the SV40 large T antigen and was cultivated in DMEM medium, 10% (v/v) FCS, penicillin (100 U/ml), and streptomycin (100 mg/ml).

### Mice

All animals were used according to protocols approved by the Institutional Animal Use Committee of the State North Rhine-Westphalia (Germany) and maintained in pathogen-free conditions in a barrier facility. C57/BL6 mice at the age of 6–8 weeks were obtained from Charles River Laboratories (Wilmington, Massachusetts, USA).

### Bacterial expression constructs

The GLuc-FimH construct encodes the Gaussia luciferase linked to the FimH protein derived from *E. coli*. The DNA gblock (Integrated DNA Technologies (IDT), Inc., Skokie, Illinois, USA) encoding the GLuc-FimH fusion protein was synthetically engineered and integrated into the bacterial expression vector pET11d (Agilent, Santa Clara, CA, USA) at the *NcoI* and *BamHI* sites. The DNA sequences encoding EGF, TGF-α and EPRG, respectively, were linked to the *E. coli* FimH DNA and the c-Myc tag for detection, a cleavage site sequence for the HRV3C mediated proteolytic release of a POI at the 3′-terminus, and flanked by the restriction sites for *NcoI* at the 5′-terminus and *BamHI* at the 3′-terminus. The corresponding gblocks were integrated into the MCS of the bacterial expression plasmid pET11d by using the restriction enzymes *NcoI* and *BamHI*, respectively. The pET11d expression vector (Agilent) provides IPTG-mediated de-repression of the T7 promoter in addition to IPTG-induction of T7 polymerase from the lacUV5 promoter in the DE3 containing *E. coli* strain BL21 (Agilent).

### Fluorescence-activated cell sorter analysis (FACS) of genetically modified *E. coli* cells

*E. coli* cells containing the plasmids pET11d-GLuc-FimH, pET11d-EGF-FimH, pET11d- TGF-α-FimH and pET11d-EPRG-FimH, respectively, were grown at 37 °C for 9 h in LB medium (10 ml) supplemented with ampicillin (200 µg/ml) and finally cultivated for additional two hours in the presence of 1 mM IPTG (Sigma-Aldrich, St. Louis, Missouri, USA) for POI expression. Bacteria were harvested, washed twice with PBS and resuspended in PBS to constitute a stock suspension with a final concentration of 1.0 at OD600nm (optical density). For the detection of GLuc on the bacterial surface, 100 µl of the bacterial suspension was incubated for 30 min with the rabbit GLuc-specific IgG serum (1:1,000) and for detection with the Phycoerythrin (PE)-conjugated anti-rabbit IgG antibody (1.0 µg/ml), clone Poly4064 (BioLegend, San Diego, CA, USA). The detection of the EGFR ligands EGF, TGF-α and EPRG on the bacterial surface was performed by the FITC-conjugated c-Myc-specific antibody, clone SH1-26E7.1.6 (Miltenyi Biotec GmbH, Bergisch Gladbach, Germany), respectively. Isotype-matched antibodies of irrelevant specificity were used as controls. Cells were analyzed on a FACSCalibur (BD Biosciences); data were analyzed using the FlowJo software (FlowJo LLC, Ashland, OR, USA).

### Immunogold electron microscopy

Wild-type and transformed *E. coli* bacteria were cultivated for 12 h at 37 °C in the presence of 1 mM IPTG in LB-medium supplemented with ampicillin (200 µg/ml) until the suspension reached an OD600nm of 1.0. The bacteria were harvested, washed twice in PBS and fixed for 1 h at room temperature by incubation with 3% (w/v) paraformaldehyde and 0.2% (w/v) glutaraldehyde in 0.1 M phosphate buffer, pH 7.4. For GLuc detection, bacteria were incubated with rabbit anti-Gaussia luciferase antibody (New England Biolabs) and gold labeled polyclonal anti-rabbit IgG antibodies (Sigma-Aldrich) according to manufacturer’s instructions. The suspension was washed three times in PBS, 1% (w/v) BSA and fixed in 3% (w/v) glutaraldehyde for 30 min at room temperature. For electron microscopy bacteria were pelleted in melting agar, dehydrated with alcohol, and embedded according to standard procedures. Sections of the material were examined with a Philips CM 10 transmission electron microscope (Philips, Amsterdam, Netherlands).

### Bioluminescence imaging

For bioluminescence imaging, GLuc modified *E. coli* (10^6^/100 µl) were cultivated on 96-well in LB medium (100 µl/well) for two hours in the presence of IPTG (1 mM), washed twice with PBS and incubated with benzyl-coelenterazine (5.0 µg/ml) for 10 minutes. Emitted photons were recorded by the Photon Imager bioluminescence device (Biospace Lab, Paris, France) equipped with the Photo Vision software (BioSpace). Non-modified *E. coli* and GLuc modified *E. coli* without IPTG induction served as controls.

### Immunocompetent mouse model of pancreas adenocarcinoma

CEAtg C57BL/6 mice were obtained from the Patterson Institute, Manchester, UK. Murine pancreatic tumors with CEA and click beetle (CB) Luc expression were induced in 10-week-old C57bl/6 mice by intra-pancreatic injection of 2 × 10^5^ Panc02 cells. For *in vivo* bioluminescence imaging, Panc02 cells were genetically modified with the CBLuc and cotransfected with the hygromycin B phosphotransferase gene for clonal selection. The tumor progression was monitored by the Photon Imager bioluminescence device (Bispace Lab) after intraperitoneal administration of the CBLuc-specific substrate D-Luc (150 mg/kg) (PJK GmbH, Kleinblittersdorf, Germany). For *in vivo* imaging, GLuc modified *E. coli* were intravenously injected into tumor bearing mice. At day 2 and day 10 upon *E. coli* inoculation, mice were treated with IPTG (1 mM) for the induction of the GLuc-FimH expression; two hours later mice were inoculated with benzyl-coelenterazine (100 µg/mouse) and analyzed for bioluminescence by the Photon Imager bioluminescence device (Biospace Lab). Bioluminescence signals were accordingly filtered against background noise. Regions of interest were defined as regions above threshold and automatically gated by predefined program tools. There was no manual gating of regions of interest in order to avoid any incoherence. Photon emission intensity (photon/s/sr) was calculated from data of emitted photons from the respective regions of interest using the M3 vision software (Biospace Lab).

### Immunohistological analyses

To detect GLuc expressing *E. coli* in tumor tissues, cryostat sections were stained with an anti-*E. coli*-HRP antibody (1.0 µg/ml) (Abcam, Cambridge, United Kingdom) to detect the bacteria and with a rabbit anti-GLuc antibody (New England Biolabs) and a HRP-conjugated anti-rabbit IgG-specific secondary antibody (Abcam) to detect GLuc. Specific binding was visualized by adding the DAB chromogen substrate (Biozol, Eching, Germany). Sections were additionally stained with H&E (Carl Roth, Karlsruhe, Germany).

### ELISA

*E. coli* were transformed with the respective plasmid encoding EGF, TGF-α and EPRG, respectively, were cultivated in LB medium for two hours in the presence of IPTG (1 mM). Bacteria pellets were resuspended in 1 ml PBS and treated with HRV 3C protease (10 U) overnight at 4 °C in order to release the POI from the FimH part of the fusion protein. Released POIs were concentrated by using of an ultra-0.5 centrifugal Amicon device (Merck KGaA, Darmstadt, Germany). The detection of the proteins of interest was performed by using ELISA kits specific for human EGF, TGF-α and EPRG, respectively (CUSABIO BIOTECH, Houston, TX, USA).

*E. coli* produced human EGF, TGF-α and EPRG, were tested for binding to EGFR. Therefore, microtiter wells were coated with released EGFR ligands (1.0 µg/ml) in PBS and incubated with human EGFR-hIgG and as control with mouse EGFR-hIgG (each 1.0 µg/ml, R&D Systems, Minneapolis, MN, USA), respectively. A human IgG antibody of irrelevant specificity served as control. Bound EGFR-IgG fusion protein was detected by an IgG-specific biotin-labeled antibody (BioLegend) and streptavidin-HRP (Sigma-Aldrich). Specific binding was visualized by using of 3,3′,5,5′-tetramethylbenzidine (TMB) substrate (Thermo Fisher Scientific) and analyzed by using of the Multiscan GO ELISA reader (Thermo Scientific); all measurements were performed in triplicates.

### EGFR phosphorylation assay

Supernatants containing the cleaved EGFR ligands EGF, TGF-α and EPRG, respectively, were quantified by ELISA and co-incubated (1.0 µg/ml) with 293T cells (10^6^ cells) in 50 µl RPMI medium. Phosphorylation of the EGFR receptor at tyrosine 1173 was detected by flow cytometry using the goat anti-human pEGFR (Tyr 1173) polyclonal antibody (Santa Cruz Biotechnology, Dallas, Texas, USA) and an APC-labeled anti-goat IgG antibody (R&D Systems, Minneapolis, MN, USA).

### Statistical analysis

Data were analyzed using GraphPad InStat, version 3.06 (GraphPad Software, San Diego, CA, USA), using unpaired or paired Student’s t test with Welch’s correction or unpaired Mann-Whitney test (for non-Gaussian distribution); p < 0.05 was considered to be significant.

### Tumor induction

All mouse studies were performed in accordance with institutional and federal guidelines (Agency for Nature, Environment and Consumer Protection of the State North Rhine-Westphalia [LANUV] registration no. 84-02.04.2012.A417).

## Supplementary information

Supplementary Figure 1
