# Supplementary material for: FimH-based display of functional eukaryotic proteins on bacteria surfaces
Source: Sci Rep. 2019 Jun 10;9:8410. doi: 10.1038/s41598-019-44883-z (PMC6557881; doi:10.1038/s41598-019-44883-z)
Supplement: Supplementary file 1 — Supplementary Figure 1 [file 41598_2019_44883_MOESM1_ESM.pdf]

SUPPLEMENTARY MATERIAL

FimH-based display of functional eukaryotic proteins on bacteria surfaces

Authors: Markus Chmielewski[1,2], Johannes Kühle[1,2], Danuta Chrobok[1,2], Nicole Riët[1,2], Michael Hallek[2] and Hinrich Abken[1,2,3]

1 Center for Molecular Medicine Cologne (CMMC), University of Cologne, Cologne, Germany

2 Department I Internal Medicine, Medical Faculty, University of Cologne, Cologne, Germany.

3 RCI, Regensburg Center for Interventional Immunology, Chair Gene-Immunotherapy, University Hospital Regensburg, Regensburg, Germany.

Supplementary Figure:

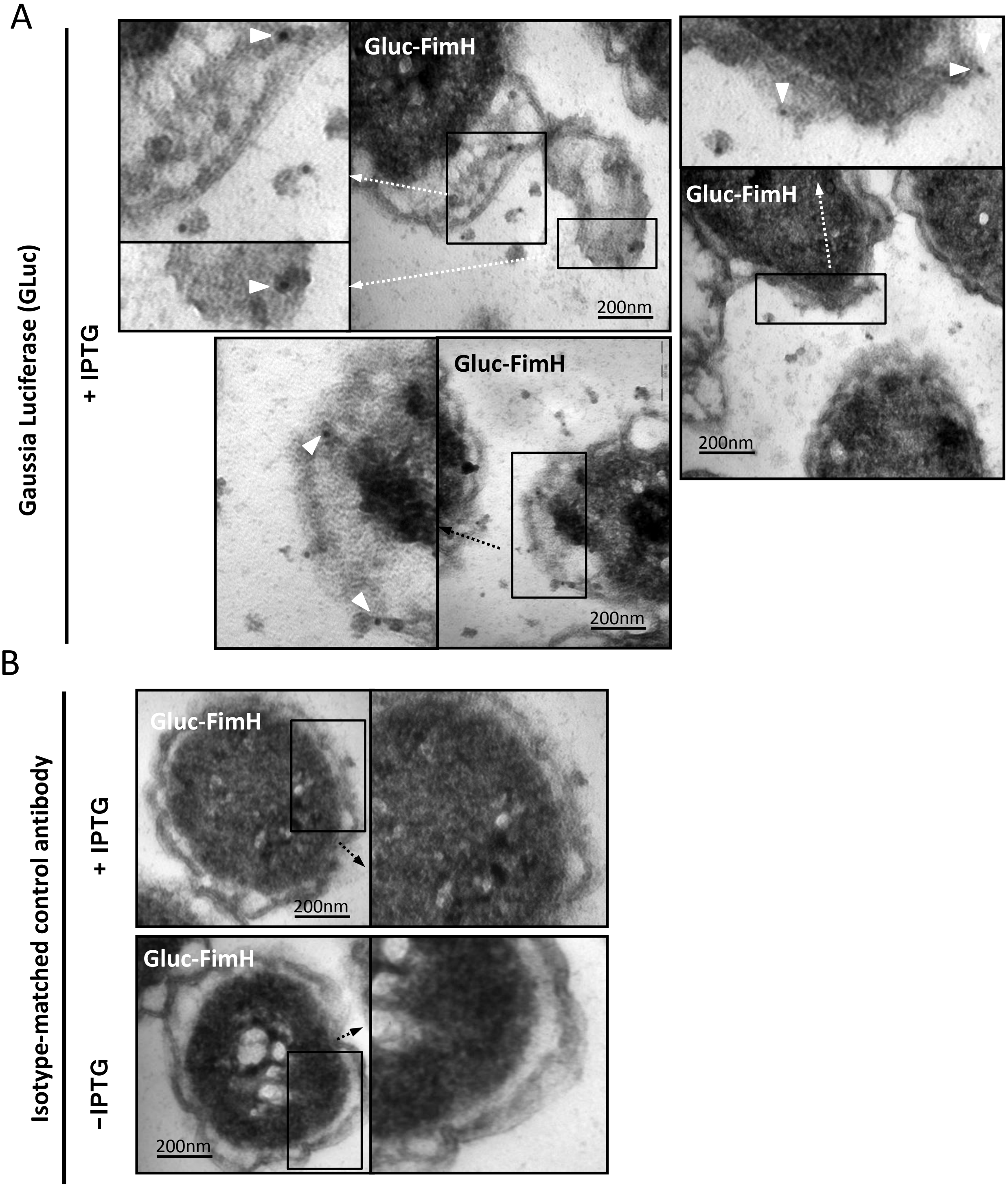

Supplementary Figure 1 (S1) Display of GLuc on the surface of genetically modified E. coli bacteria. (A) GLuc-FimH modified E.coli bacteria were incubated in the presence of IPTG for GLuc expression. The samples were fixed and the expression of GLuc-FimH on the bacterial surface was detected by immunogold staining. White arrowheads indicate the presence of GLuc on the bacteria surface. (B) The specificity of performed immunogold staining was confirmed by using an isotype-matched control antibody.
